# Cancer during pregnancy alters the activity of rat placenta and enhances the expression of cleaved PARP, cytochrome-c and caspase 3

**DOI:** 10.1186/1471-2407-6-168

**Published:** 2006-06-26

**Authors:** Mércia Tancredo Toledo, Gislaine Ventrucci, Maria Cristina Cintra Gomes Marcondes

**Affiliations:** 1 Departamento de Fisiologia e Biofísica, Instituto de Biologia, Universidade Estadual de Campinas (UNICAMP), Campinas, CP 6109, 13083-970, SP, Brazil

## Abstract

**Background:**

The presence of cancer makes it difficult to predict the progress of pregnancy and can be deleterious to the maternal-foetal relationship. Apoptosis may affect a range of placental functions and result in the retardation of foetal growth. In this work, we investigated the placental alterations produced by tumour growth and the effects on the expression of apoptotic factors in placental tissue.

**Methods:**

Adult female Wistar rats (90 days old, n = 54) were allocated to control (C), tumour-bearing (W), or ascitic fluid-injected (A) groups and were killed on the 16^th^, 19^th ^or 21^st ^day of pregnancy. Placental tissues were analysed using biochemical and histochemical assays.

**Results:**

The placental protein content and glutathione-S-transferase activity were decreased in groups W and A. Histochemical analysis showed an increase in the number of cells with cleaved PARP, caspase 3 and cytochrome-c in groups W and A, indicating that the tumour growth clearly damaged placental tissue and affected the levels of apoptotic factors. These results were confirmed by western blotting.

**Conclusion:**

Since trophoblastic cells are responsible for maintaining a normal placental function, the uncontrolled death of these cells in response to tumour cell growth or substances derived from ascitic fluid could have a negative impact on foetal development. Further knowledge of these events may help to preserve the foetus and placenta during development.

## Background

Cancer is the second most common cause of death during the reproductive years [1] and complicates 0.02–0.1% of all pregnancies [2]. However, the development of cancer during pregnancy is difficult to predict [3]. Indeed, some studies have suggested that pregnancy does not favour the development of cancer and may protect the organism against tumour growth [3,4]. Fast tumour growth during pregnancy may result in damage to the foetus and lead to foetal resorption and death [4-6]. Since foetal and tumour growth requires increased protein synthesis, the importance of amino acids in foetal life has been emphasised [7].

The foetal nutrient supply depends on the mother's reserves and food intake, as well as on the placental function. Placental growth increases during gestation, concomitant with or ahead of foetal growth [8], and a failure in placental function can adversely affect foetal growth or welfare [7]. The physiological changes that occur during pregnancy can only be sustained if there is an appropriate nutrient supply to ensure placental and foetal development [7]. To guarantee the supply of nutrients essential to foetal survival, the placenta continuously undergoes changes in weight, structure, shape and function during gestation [9].

The placenta-foetal metabolic relationships are complex, dynamic processes that control many aspects of foetal development. The exchange between maternal and foetal blood occurs across the labyrinth layers of the placental barrier. In rats, the placenta, which consists of three trophoblast layers in the labyrinth zone, is the rate-limiting permeability barrier to substrate exchange between the maternal and foetal compartments [10].

Programmed cell death has been implicated in normal and pathological processes in several human tissues and diseases, including cancer [11,12]. Placental apoptosis occurs during normal pregnancy [13,14], especially in trophoblast cells, whereas excessive apoptosis of the syncytiotrophoblast and cytotrophoblast, including the extravillar population, has been observed in pre-eclampsia pregnancies [16-18]. Recently, a number of studies have suggested that apoptosis plays a role in the normal development, remodelling, and ageing of the placenta [15,19,20], particularly in the second half of pregnancy [15].

During apoptosis, the activation of caspases by nuclear, metabolic or external stimuli occurs in a cascade fashion, and results in nuclear engulfment and cell death [21,22]. Since apoptosis may affect a range of placental functions, and since an increase in this process may be associated with the retardation of foetal growth, we investigated the influence of Walker 256 carcinosarcoma growth on the organisation of the placenta in rats and on the presence of apoptotic signals, such as cleaved PARP, cytochrome-c, and caspase 3.

## Methods

### Animals

Adult female Wistar rats (90 days old, n = 54) were obtained from the animal facilities of the State University of Campinas (CEMIB-UNICAMP) and were housed with adult males (four females per male) for 12 h [23]. Mating was confirmed by analysing vaginal smears, with a positive result being considered as the first day of pregnancy. The pregnant rats were allocated to one of three groups: control (C), tumour-bearing (W), or injected with ascitic fluid (A). All of the rats were housed in collective cages under standard conditions (22 ± 2°C, 12 h light-dark cycle) with free access to a semi-synthetic control diet (AIN-93 G) [24].

### Neoplastic implants and extraction of ascitic fluid

Walker 256 carcinosarcoma cells (2.5 × 10^5 ^in approximately 0.5 mL of 0.9%, w/v, NaCl), originally obtained from the Arthur D'Little Hospital, USA, were injected subcutaneously on the second day of pregnancy. Control rats received 0.5 ml of 0.9% NaCl solution. Since we have previously shown that ascitic fluid from tumour-bearing rats produces foetal death and resorption [4], we examined the effect of this fluid on tumour growth. Ascitic fluid was collected from the intraperitoneal cavity of Walker tumour-bearing rats and was centrifuged (500 × *g*, 4°C, 10 min) to remove all neoplastic cells. From the 9^th ^day of pregnancy onwards, group A rats received daily injections of 2 ml of ascitic fluid. The rats were killed 16, 19 or 21 days after mating, which corresponded to the 14^th^, 17^th ^and 19^th ^day after tumour transplantation, or the 7^th^, 10^th ^and 12^th ^day after ascitic fluid injection, respectively.

The general UKCCR (United Kingdom Co-ordinating Committee on Cancer Research) guidelines for animal welfare [25] were followed and the protocols were approved by the institutional Committee for Ethics in Animal Experimentation (CEEA/IB/UNICAMP, protocol #217-3].

### Histochemical analyses

The whole placental tissue from all rats were processed for light microscopical immunohistochemistry after perfusing the uterus, via the heart, with 10 mL of heparinized saline followed by 20 mL of fixative solution containing 4% paraformaldehyde in phosphate-buffered saline (PBS), pH 7.2. The fixed placentas were then removed, dehydrated, and embedded in paraffin. Sections (at least 5 μm thick) were obtained for the immunohistochemical detection of cleaved PARP, caspase 3 and cytosolic cytochrome-c.

Placental cells were detected immunohistochemically using anti-PARP [poly(ADP-ribose) polymerase] goat polyclonal antibody (Santa Cruz Biotechnology, Santa Cruz, CA; diluted 1:100) followed by incubation with biotinylated rabbit anti-goat secondary antibody (1:500 dilution, Santa Cruz Biotechnology) for 1 h at room temperature and further incubation with streptavidin horseradish peroxidase solution (Vector Laboratories). The sections were reacted with diaminobenzidine (DAB, Sigma) and H_2_O_2 _in PBS and stained with fast green. Caspase-3 and cytochrome-c were detected using specific goat polyclonal antibodies (Santa Cruz Biotechnology; diluted 1:250 and 1:100, respectively) and the biotinylated secondary antibody/streptavidin horseradish peroxidase detection system described above. After incubation with DAB and H_2_O_2 _in PBS, the sections were stained with Harris' haematoxylin and lithium carbonate. For negative controls, placental sections from each group were incubated in PBS without the first antibody and then incubated with the biotinylated anti-goat secondary antibody followed by reaction with DAB, as described above.

The cells positive for cleaved PARP, caspase-3 and cytosolic cytochrome-c were counted using Image Pro-Plus software (v.3.01, Media Cybernetics, Silver Spring, MD, USA) after capturing the image on a Leica microscope (Leica DMLM, Weizlar, Germany) using a 100× magnification for cleaved PARP images and a 40× magnification for caspase-3 and cytochrome-c images. The number of positive cells was determined by counting 20 fields (300 μm^2 ^each) in one slide from each of at least six dams per group.

### Biochemical assays and Western blotting

The other portion of the uterus from all groups was used to provide placental tissue for biochemical assays and western blotting. Fragments of this tissue were stored at -70°C until analysis. Placental samples (100 mg) were homogenised in buffer (1:4, w/v, in 0.3 M Tris pH 8, 0.1 M NaCl, 10 mM EDTA, 0.2 M sucrose and 0.01% SDS) and centrifuged at 10,000 × *g *for 15 min at 4°C. The total protein content of the supernatants was determined colorimetrically as described by Bradford [26]. The homogenates were used to determine the glutathione-S-transferase activity with the assay described by Habig et al. [27]. Western blotting was used to assess changes in the expression of cleaved PARP, caspase 3 and cytochrome-c after SDS-PAGE of 20 μg of total protein in 12% polyacrylamide gels. For western blots of cytochrome-c, the placental homogenates were subjected to three freeze-thaw cycles to obtain a cytosolic fraction without mitochondria prior to electrophoresis. The proteins were transferred to 0.45 μm nitrocellulose Hybond membranes (Amersham Biosciences, Piscataway, NJ) and then incubated with polyclonal antibodies against cleaved PARP (Santa Cruz Biotechnology; diluted 1:500) and caspase 3 (Santa Cruz Biotechnology; diluted 1:1000) or a monoclonal antibody against cytochrome-c (R&D, diluted 1:1000), followed by detection with an anti-goat secondary horseradish peroxidase (HRP)-labelled antibody (Dako, Carpinteria, CA, USA, diluted 1:1500). Actin was used as an internal control for protein loading and was detected with an antibody to mouse actin. The nitrocellulose membranes were developed using enhanced chemiluminescence (ECL reagent, Amersham Biosciences) and densitometric analysis of the protein bands was done using Gel-Pro Analyser software (Media Cybernetics, 1993–97, Silver Spring, MD, USA).

### Statistical analysis

The results were expressed as the mean ± SEM. Inter- and intragroup comparisons were done using one-way analysis of variance (ANOVA) [28] followed by Dunn's multiple comparison tests to determine the significance of the differences among the groups. All statistical comparisons were done with GraphPad Prism software (v 3.0, GraphPad Inc., San Diego, CA, USA), with a value of P < 0.05 indicating significance.

## Results

### Influence of Walker 256 tumour and ascitic fluid on foetal and placental parameters

**Figure 1:**
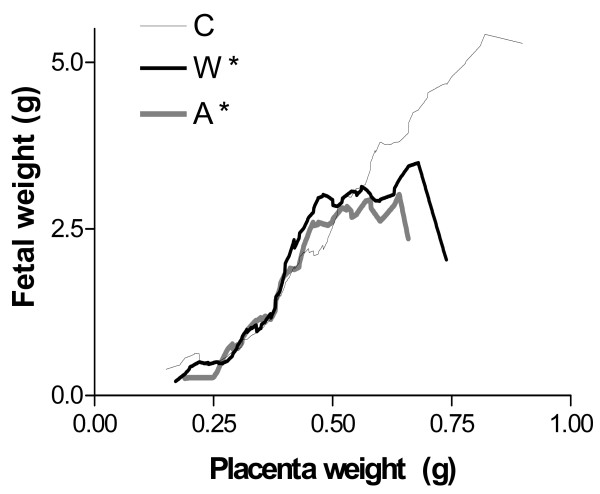
The foetal and placental ratios of the control, tumour-bearing and ascitic fluid-injected groups. The foetal weight and corresponding placental weight were plotted and the positive correlation between these parameters was analysed using GraphPad Prism software. There was a significant difference (*P < 0.05) between the tumour-bearing and ascitic fluid-injected groups compared to the control rats. Groups: C – control rats, W – tumour-bearing rats and A – rats injected with ascitic fluid. The rats in each group were sacrificed on the 16^th^, 19^th ^and 21^st ^day of pregnancy.

The foetal and placental ratio was altered by tumour growth and the injection of ascitic fluid. Figure 1 shows the reduction in the foetal and placental ratio in groups W and A, with different correlations for each curve (P < 0.05).

The placental protein levels of the control groups were similar, regardless of the gestational stage (C16 = 2.23 ± 0.11, C19 = 2.22 ± 0.12 and C21 = 2.19 ± 0.10 μg/μL; n = 8 each). In the tumour groups, the protein content was significantly lower (P < 0.05) than in the corresponding control only for W21 (W16 = 2.12 ± 0.16, W19 = 2.15 ± 0.06 and W21 = 1.64 ± 0.18 μg/μL; n = 8 each). A significant decrease in the protein content (~27%; P < 0.05) was seen in all groups injected with ascitic fluid (A16 = 1.38 ± 0.09, A19 = 1.59 ± 0.09 and A21 = 1.58 ± 0.08 μg/μL; n = 8 each).

Glutathione-S-transferase (GST) activity is associated with the cell cycle and is responsible for the conjugation and detoxification of intermediates produced by oxidative stress in rat placenta [29]. There was a non-linear decrease in GST activity during pregnancy in the control groups (C16 = 23.94 ± 1.83, C19 = 31.71 ± 4.28*, and C21 = 14.89 ± 1.31* nmol/μg protein, n = 8; *P < 0.05 compared to the C16 group), since the high GST activity seen on the 19^th ^day may be related to the maximal placental function at this time interval, as previously reported [30]. Tumour-bearing rats showed a significant decrease in GST activity on days 19 and 21 when compared to the corresponding control groups (W16 = 21.23 ± 2.15, W19 = 20.47 ± 1.94 and W21 = 9.23 ± 1.84 nmol/μg protein; n = 8; P < 0.05). These results indicated an attenuation of the cellular protective mechanisms during tumour growth that could possibly compromise placental function and affect foetal development, as previously demonstrated [4,30].

Similar data were obtained in the ascitic fluid group, in which GST activity was reduced on the 19^th ^and 21^st ^days (A16 = 17.73 ± 2.65, A19 = 13.94 ± 1.50 and A21 = 11.15 ± 1.17 nmol/μg protein, n = 8; P < 0.05) compared to the control group. These data suggest that the presence of ascitic fluid decreases the protective action of anti-oxidative enzymes such as GST.

### Influence of Walker 256 tumour and ascitic fluid on the immunohistochemical detection of placental cleaved PARP, cytochrome-c and caspase-3

**Figure 2:**
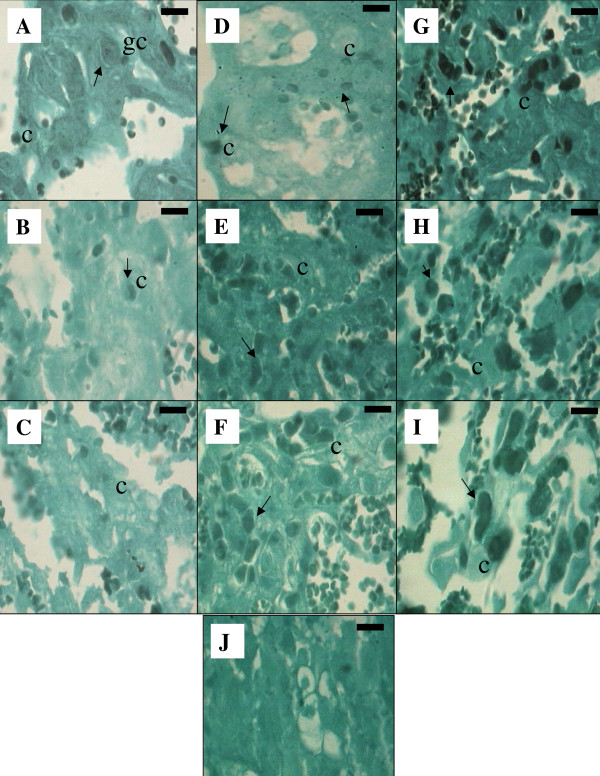
Immunohistochemical detection of cleaved PARP in placental tissue at different stages of pregnancy (days 16, 19 and 21). Cleaved PARP was detected using a polyclonal antibody (Santa Cruz; diluted 1:100) followed by incubation with a biotinylated anti-goat antibody (1:500) and reaction with DAB in the presence of H_2_O_2_. The tissue sections are from the placental junctional zone, which contains cytotrophoblastic cells (c) and trophoblastic giant cells (gc). The arrows indicate nuclei with immunostaining for cleaved PARP. Groups: C – control rats, corresponding to images A, B and C on the 16^th^, 19^th ^and 21^st ^day, respectively, W – tumour-bearing rats, corresponding to images D, E and F on the 16^th^, 19^th ^and 21^st ^day, respectively, and A – rats injected with ascitic fluid, corresponding to images G, H and I on the 16^th^, 19^th ^and 21^st ^day, respectively. Image J is a negative control. Magnification – 100×. Horizontal bar = 100 μm.

The placental histological layer investigated here was the junctional zone, which contains cytotrophoblastic cells, trophoblastic giant cells and vesicular glycogen cells [31] (see Figure 2A for a placental section from a control rat on the 20^th ^day of pregnancy). The labyrinth zone is not shown since the alterations in this zone were the same as those in the junctional zone

PARP is a nuclear protein implicated in DNA repair and is the earliest protein cleaved to a specific 89 kDa fragment (cleaved PARP) during apoptosis [32]. Immunohistochemical analysis for cleaved PARP revealed few stained nuclei in sections from the control groups (Figure 2A–C). However, in the nuclei that were positive, the staining was strong, especially in cytotrophoblastic and trophoblastic giant cells. Compared to the corresponding controls, there was a significant increase in the number of nuclei positive for cleaved PARP in tumour-bearing rats (W16, W19 and W21), regardless of the gestational stage (Figure 2D,E and 2F, respectively). The ratio of positive cells (those with stained nuclei) in the W and C groups showed that the level of cleaved PARP increased by about three-fold, regardless of the gestational stage (Table 1). Similar results were obtained in all of the ascitic fluid groups, with strong nuclear staining, particularly in cytotrophoblastic and trophoblastic giant cells (Figure 2G,H, and 2I, which correspond to A16, A19 and A21, respectively). The ratio of positive cells in the ascitic fluid and control groups indicated an ~1.5-fold increase in the former group compared to the controls, but this increase was not as high as in the W group, except on the 19^th ^day of pregnancy (Table 1).

**Figure 3:**
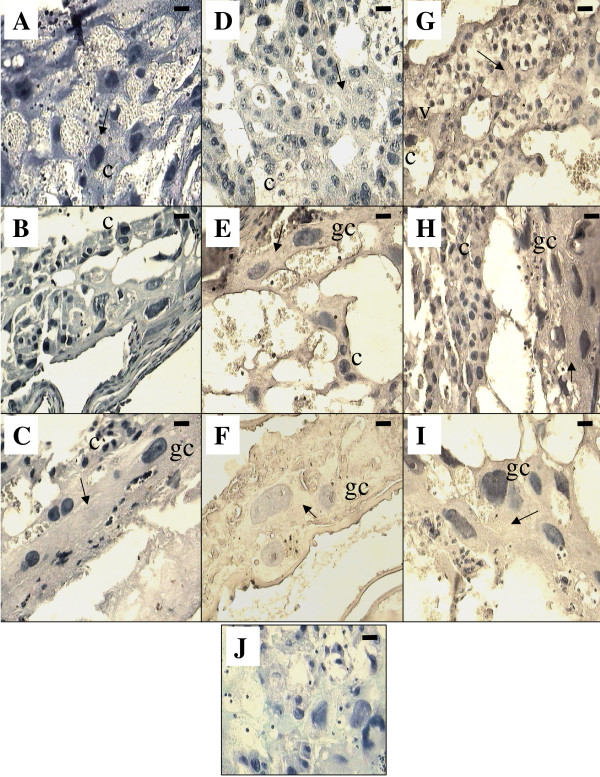
Immunohistochemical detection of cytosolic cytochrome-c in placental tissue in different stages of pregnancy (days 16, 19 and 21). Cytochrome-c was detected using a polyclonal antibody (Santa Cruz Biotechnology, diluted 1:100) followed by incubation with a biotinylated anti-goat antibody (1:500) and reaction with DAB in the presence of H_2_O_2_. The arrows indicate the cytoplasmic immunostaining of cytochrome-c in trophoblastic cells. The tissue section contains cytotrophoblastic cells (c), trophoblastic giant cells (gc) and vesicular glycogen cells (v). Groups: C – control rats, corresponding to images A, B and C on the 16^th^, 19^th ^and 21^st ^day, respectively, W – tumour-bearing rats, corresponding to images D, E and F on the 16^th^, 19^th ^and 21^st ^day, respectively, and A – rats injected with ascitic fluid, corresponding to images G, H and I on the 16^th^, 19^th ^and 21^st ^day, respectively. Image J is a negative control. Magnification – 40×. Horizontal bar = 10 μm.

Cytochrome-c showed a similar distribution in the cytosol of placental cells during different stages of pregnancy in the control groups (the number of positive cells was similar in C16, C19 and C21; Figure 3A,B, and 3C, respectively, and Table 1). In tumour-bearing rats, there was enhanced staining for cytosolic cytochrome-c, particularly in cytotrophoblastic and trophoblastic giant cells (Figure 3D,E, and 3F, corresponding to W16, W19 and W21, respectively). The cytosolic staining for cytochrome-c was disperse and enhanced in the ascitic fluid groups at all stages of pregnancy (Figure 3G,H, and 3I, corresponding to A16, A19 and A21, respectively).

**Figure 4:**
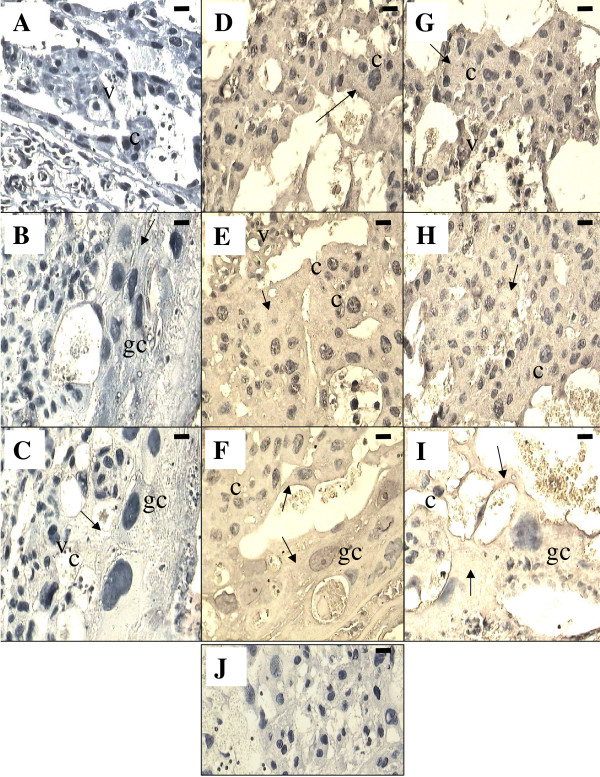
Immunohistochemical detection of caspase-3 in placental tissue in different stages of pregnancy (days 16, 19 and 21). Caspase 3 was detected using a polyclonal antibody (Santa Cruz Biotechnology, diluted 1:100) followed by incubation with biotinylated anti-goat antibody (1:500) and reaction with DAB in the presence of H_2_O_2_. The arrows indicate the cytoplasmic immunostaining of caspase-3 in trophoblastic cells. The tissue section contains cytotrophoblastic cells (c), trophoblastic giant cells (gc) and vesicular glycogen cells (v). Groups: C – control rats, corresponding to images A, B and C on the 16^th^, 19^th ^and 21^st ^day, respectively, W – tumour-bearing rats, corresponding to images D, E and F on the 16^th^, 19^th ^and 21^st ^day, respectively, and A – rats injected with ascitic fluid, corresponding to images G, H and I on the 16^th^, 19^th ^and 21^st ^day, respectively. Image J is a negative control. Magnification – 40×. Horizontal bar = 10 μm.

There was a slight increase in cytosolic staining for caspase-3 expression in the placenta of control dams at C16, the first stage of pregnancy, compared to the other days (C19 and C21) (Table 1 and Figure 4A,B and 4C, which correspond to C16, C19 and C21, respectively). Compared to control rats, the tumour-bearing rats and rats injected with ascitic fluid showed a significant increase in caspase-3 staining in the cytosol of cytotrophoblastic and trophoblastic giant cells, with positive cells in all stages of pregnancy (P < 0.05) (Figure 4D,E and 4F for group W and Figure 4G,H and 4I for group A) (Table 1). The ratio of positive cells in groups W and A compared to the corresponding controls indicated a three-fold increase in caspase-3 expression at all gestational stages, except for group W19, for which the increase was approximately seven-fold (Table 1).

### The expression of cleaved PARP, cytochrome-c and caspase 3 in rat placenta

**Figure 5:**
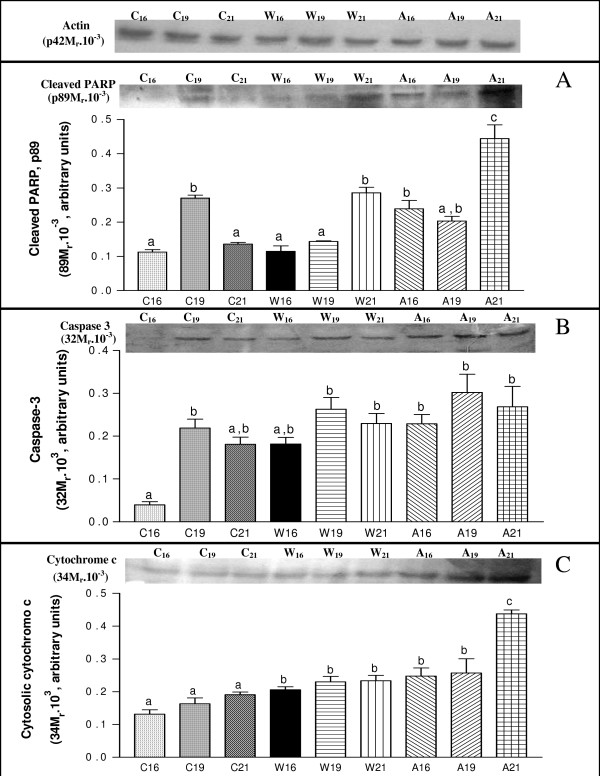
Densitometric analysis of western blots (WB) of cleaved PARP (89 kDa; A), caspase 3 (32 kDa; B) and cytosolic cytochrome-c (34 kDa; C) and WB images of cleaved PARP, caspase-3 and cytosolic cytochrome-c in the spongy trophoblast of control, tumour-bearing and ascitic fluid-injected rats on the 16^th^, 19^th ^and 21^st ^day of pregnancy. Cleaved PARP (antibody dilution 1:500), caspase-3 and cytochrome-c (antibody dilution 1:1000) were detected with polyclonal antibodies from Santa Cruz Biotechnology and enhanced chemiluminescence. For details see *Materials and Methods*. The band intensities (expressed in arbitrary densitometric units) were analysed using Gel-Pro Analyser software. Actin was used as an internal control for protein loading and was detected with an antibody to mouse actin. The WB images are representative of eight separate blots. Groups: C – control, W – tumour-bearing, and A – ascitic fluid-injected rats. The results are expressed as the mean ± SEM of at least six rats per group. Different superscript letters indicate significant differences (P < 0.05) within each column.

Western blotting showed that the expression of cleaved PARP (89 kDa band) in placental tissue was significantly enhanced on the 21^st ^day in group W and on the 16^th ^and 21^st ^days in group A; the increase on the 21^st ^day was around two- and three-fold higher in groups W and A, respectively (Figure 5A, Table 1). There is a positive correlation between gestational stage and the levels of the 89-kDa fragment and placental apoptosis during normal pregnancy [33]. Our findings indicate that there was activation on apoptosis process due to the tumour growth or the presence of ascitic fluid. The expression of cytochrome-c in the control groups was similar, regardless of the stage of pregnancy (Figure 5C). The principal death-signalling pathway involves mitochondria that, in response to various stimuli, can release cytochrome-c into the cytosol where this enzyme triggers apoptotic effectors such as caspase activation [34]. An increase in the expression of placental cytochrome-c in groups W and A, especially on the 16^th ^and 19^th ^days, with an additional increase on the 21^st ^day in rats given ascitic fluid (Figure 5C), suggested the occurrence of tissue damage caused by the tumour or by products synthesized by the tumour and/or host cells present in ascitic fluid. Western blotting revealed a tendency towards increased caspase-3 expression during pregnancy in the tumour and ascitic fluid groups, especially on the 19^th ^and 21^st ^days (Figure 5B).

## Discussion

The nutritional demand of tumour cells is extremely high [35] and may produce metabolic alterations that can compromise the availability of amino acids and other nutrients to foetal tissues [36]. Ascitic fluid contains many factors capable of causing oedema and haemorrhage in the placenta of pregnant rats [37] and of decreasing the placental glycogen stores in tumour-bearing rats [30].

An optimal maternal-foetal exchange is necessary for a successful pregnancy. Complications associated with pregnancy, including limited intrauterine growth and pre-eclampsia, have been related to poor trophoblast invasion and/or placental insufficiency that may result in placental ischemia and oxidative stress [38]. However, the mechanisms by which placental oxidative stress triggers the characteristic placental endothelial dysfunction responsible for the development of pre-eclampsia are not well understood [38]. Placental anti-oxidative mechanisms play an important role during pregnancy, as shown by the elevated activities of GSH, GST isoenzymes and GST and GPX [38]. Cervello et al. [29] studied pregnant rats treated with benzo(a)pyrene and reported an increase in the anti-oxidative capacity, seen as enhanced GST activity in the placental tissue of these rats compared to non-treated animals. However, these authors concluded that the increase in GST activity was insufficient to protect the foetus and that benzo(a)pyrene significantly increased the number of resorptions and reduced the foetal weight [29].

As shown here, there was a marked reduction in the GST activity of placental tissue in groups W and A. Assuming that tumour growth during pregnancy induces foetal resorption and death [4] and jeopardises the placental tissue by causing placental oedema [37] and a reduction in the level of placental glycogen [30], the changes in placental GST activity seen here could have an antioxidant function and may occur in parallel with programmed cell death. In late gestation in humans, there is increased oxidative stress in pregnancies complicated by diabetes, intrauterine growth retardation (IUGR) and preeclampsia, as well as increased trophoblast apoptosis and deportation, and altered placental vascular reactivity [39].

Tumour growth clearly damaged the placental tissue by altering the foetal/placental ratio and the levels of apoptotic signalling molecules such as cleaved PARP, cytochrome-c and caspase 3. Tumours can damage foetuses (high foetal resorption, foetal death or a decrease in foetal growth) through competition for nutrients and by harming the foetus and placenta indirectly through substances synthesized by the tumour and/or host cells present in ascitic fluid [4]. Apoptosis occurs during normal development in different organs and tissues and is a normal phenomenon in trophoblast turnover, with no inflammatory response in the mother. Apoptosis is important for an appropriate balance in cell turnover, with the early, reversible stages of the apoptotic cascade being involved in the differentiation and fusion of the cytotrophoblast in animal models of pregnancy [39-42].

Normal placental development depends upon the differentiation and invasion of the trophoblast, the main cellular component of the placenta. Apoptosis in the trophoblast increases in normal placentas as gestation proceeds, with a higher incidence in pregnancies complicated by preeclampsia or IUGR [41]. Apoptosis is triggered by different cell-type-specific signals that involve mitochondrial and receptor-mediated pathways and results in activation of the caspase cascade. At least two major mechanisms by which a caspase cascade may be initiated have been suggested: one involves death receptors that activate initiator caspases [43] and the other involves cytochrome-c release [44]. There is evidence for the occurrence of one of the apoptotic pathways induced by cytochrome-c in the placenta of tumour-bearing rats and in rats injected with ascitic fluid. Further studies need to address other apoptotic pathways involved during tumour growth in placenta. Hung et al. [45] reported that hypoxia-reoxygenation in vitro stimulated apoptosis in human placental tissue, and that there was a significant increase in cytochrome-c release from mitochondria associated with intense immunolabelling for active caspase 3 in the syncytiotrophoblast and foetal endothelial cells. There was also increased labelling of syncytiotrophoblastic nuclei for cleaved PARP, and higher cytosolic concentrations of cleaved PARP in placental tissue under hypoxia [45].

In agreement with these findings, we observed increased caspase 3 expression in groups W and A that may have been initiated by the release of cytochrome-c into the cytosol [46]. Indeed, the injection of ascitic fluid increases caspase 3 activity [47], which then cleaves PARP, one of many caspase substrates, thereby preventing this enzyme from repairing damaged DNA [48]; this lack of repair can lead to apoptosis. Western blotting showed an increase in cleaved PARP and caspase 3 expression in groups W and A that was also associated with a progressive enhancement of the cytochrome-c levels in both groups. These results may indicate that placental layers, especially the trophoblast, may experience greater apoptosis mediated by cytochrome-c release and caspases, including caspase-3, which is activated by several death receptors [43]. Such activation could be related to the decrease in GST activity seen in groups W and A. This would allow the formation of reactive oxygen species [49], which could in turn activate other apoptotic pathways [50,51]. Various studies have reported increased apoptosis following B19 infection of villous trophoblastic cells [52], an elevation in caspase 3 activity and cytochrome-c release in chorionic villi after exposure to ultrasound [53], and higher apoptotic rates in placentas from pregnancies complicated with intrauterine growth restriction [54].

## Conclusion

Since trophoblastic cells are responsible for maintaining a normal placental function, the association to tumour growth and/or substances produced by tumour cells, such as cytokines and factors present in ascitic fluid, could be involved in the uncontrolled death of trophoblastic cells that have a negative impact on foetal development. Further work are underway in our laboratory to improve our understanding of the events that occur at the maternal-foetal interface in the presence of tumours, especially with regard to the role of cytokines present in ascitic fluid. Such knowledge may help to improve the welfare of the foetus and placenta during tumour development.

## Competing interests

The author(s) declare that they have no competing interests.

## Authors' contributions

Author 1 M.T.T. carried out the experiments, biochemical and immunohistochemical assays and performed the statistical analysis.

Author 2 G.V. carried out the biochemical assays and performed the statistical analysis.

Author 3 M.C.C.G.M. conceived of the study and participated in its design and coordination.

All authors read and approved the final manuscript.

## Pre-publication history

The pre-publication history for this paper can be accessed here:



## Figures and Tables

**Table 1:** Placental immunohistochemical analysis of cleaved PARP, caspase-3 and cytosolic cytochrome-c (number of positive cells counted) and the ratios of cleaved PARP, caspase-3 and cytosolic cytochrome-c positive cells in the spongy trophoblast layer of tumour-bearing rats or rats injected with ascitic fluid compared to control rats at different stages of pregnancy (16^th^, 19^th ^and 21^st ^days). Polyclonal antibodies against PARP, caspase-3 and cytochrome-c (all from Santa Cruz Biotechnology; anti PARP and anti-cytochrome-c diluted 1:100; anti-caspase diluted 1:250) were detected with a secondary biotinylated anti-goat antibody (diluted 1:500) and reacted with DAB in the presence of H_2_O_2_. The cells were counted using Image ProPlus software.

**Number of positive cells counted**				**Ratio between treated and control groups**			
	**Cleaved PARP**	**Caspase-3**	**Cytosolic cytochrome-c**		**Cleaved PARP**	**Caspase-3**	**Cytosolic cytochrome-c**

**C16**	43.1 ± 7.1^a^	3.7 ± 0.4^a^	3.4 ± 0.3^a^				
**W16**	136.7 ± 12.2^b^	16.0 ± 1.4^c^	4.7 ± 0.3^a^	**W16/C16**	2.89 ± 0.30^a^	3.02 ± 0.37^a^	1.52 ± 0.11^a^
**A16**	71.7 ± 9.2^c^	7.9 ± 0.4^c^	6.7 ± 0.4^c^	**A16/C16**	1.41 ± 0.13^b^	2.90 ± 0.35^a^	2.54 ± 0.25^b^
**C19**	48.0 ± 5.9^a^	1.3 ± 0.2^b^	4.9 ± 0.3^a^				
**W19**	149.2 ± 20.9^b^	9.3 ± 0.7^c^	13.3 ± 0.7^b^	**W19/C19**	3.40 ± 0.23^a^	7.56 ± 0.74^b^	3.03 ± 0.20^b^
**A19**	115.9 ± 9.5^b^	4.6 ± 0.2^a^	15.4 ± 0.4^d^	**A19/C19**	3.00 ± 0.30^a^	3.84 ± 0.34^a^	3.35 ± 0.21^b^
**C21**	54.8 ± 7.6^a^	2.5 ± 0.3^a^	4.8 ± 0.3^a^				
**W21**	148.0 ± 16.9^b^	7.3 ± 0.7^c^	12.7 ± 0.6^b^	**W21/C21**	2.99 ± 0.54^a^	2.50 ± 0.70^a^	3.00 ± 0.22^b^
**A21**	73.4 ± 11.7^c^	7.2 ± 0.4^c^	10.2 ± 0.6^b^	**A21/C21**	1.67 ± 0.36^b^	2.43 ± 0.50^a^	2.78 ± 0.29^b^

Groups: control (C), tumour-bearing (W) and ascitic fluid-injected (A) rats. Twenty areas (300 μm^2 ^each) were examined in one tissue section each from at least six rats per group. The cells were counted using Image ProPlus software. The results are expressed asthe mean ± SEM. Different superscript letters indicate significant differences within each column (P < 0.05).
